# Reproductive outcomes of vitrified blastocyst transfer in modified natural cycle versus mild hormonally stimulated and artificial protocols: A randomized control trial

**DOI:** 10.5935/1518-0557.20180040

**Published:** 2018

**Authors:** Otoufe Sheikhi, Masoumeh Golsorkhtabaramiri, Sedighe Esmaeilzadeh, Treza Mahouti, Fateme Nadi Heidari

**Affiliations:** 1 Infertility and Reproductive Health Research Center, Health Research Institute, Babol University of Medical Science, Iran

**Keywords:** Pregnancy outcome, natural cycle, blastocyst, vitrification

## Abstract

**Objective:**

This study set out to investigate the pregnancy outcome of natural cycle regimen versus other endometrial preparation protocols with vitrification thawed blastocyst transfer (VTBT) cycles.

**Methods:**

This control trial study was carried out on 123 women undergoing VTBT. The women were randomly divided into three groups of endometrial preparation before VTBT; 1. Modified natural ovulation cycle with using HCG (n=32) 2. Mild hormonally stimulated cycle by low dose Clomiphene Citrate (n=30) and 3. Artificial cycle induced with estradiol and progesterone supplementation (n=61). Following endometrial preparation, the thawed blastocyst was vitrified and transferred. Reproductive outcome and endometrium characteristic were evaluated in the three groups.

**Results:**

The three above-mentioned protocols resulted in clinical pregnancy rates of 21.43% *vs.* 13.79% *vs.* 15.25%, respectively; without statistical differences. The ongoing pregnancy rates did not show any significant differences among the three groups (21.43% *vs.* 13.79% vs. 13.56%), respectively. In addition, the miscarriage rates were compared in the three groups. The endometrial thickness on the day of progesterone or human chorionic gonadotropin administration were more frequently observed in the artificial and modified natural cycle versus hormonally stimulated groups (8.34±0.89 vs. 7.3±1.4, *p*<0.001; 8.13±0.95 vs. 7.3±1.4, *p*<0.001). There was no significant difference regarding triple-line endometrial patterns in the three groups.

**Conclusion:**

The natural cycle with HCG trigger could be considered as an alternative protocol to mild hormonally or artificial cycle regimens in vitrification thawed blastocyst transfers.

## INTRODUCTION

A good proliferative modification of endometrium is a key factor to nourish a blastocyst in assisted reproduction cycles. Endometrium thickness with or without the endometrial pattern is the outstanding sonographic parameter that have been widely used to obtain maximal endometrial receptivity (Kasius *et al*., 2014; Arce *et al*., 2015; Yoeli *et al*., 2004; Zhao *et al*., 2012; Hancke *et al*., 2012; Kovacs *et al*., 2003). A variety of endometrial maturation regimens has been investigated, to improve endometrium receptivity. Artificial cycle regimen is the most common protocol for hormone replacement therapy for endometrium preparation prior to blastocyst transfer. In this routine hormone replacement therapy protocols, the endometrium is induced by exogenously administered estradiol and progesterone (Veleva *et al*., 2013; Lathi *et al*., 2015; Morozov *et al*., 2007; Wright *et al*., 2006). Estrogen is used until the endometrial thickness on ultrasound meets approximately 0.8cm (Singh *et al*., 2011), then, progesterone initiates according to the stage of blastocyst development. Progesterone stimulation for a specific number of days will induce endometrial receptivity (Paulson, 2011).

To date, one of the other common methods for endometrium preparation is adding GnRH to hormone supplements, or with follicle-stimulating drugs such as Clomiphene Citrate (Gelbaya *et al*., 2006; El-Toukhy *et al*., 2004; Arefi *et al*., 2016; Peeraer *et al*., 2015). Modification of physiologic endometrium concentration, the concern of hormonal exposure in the uterus, greater drug doses required, hormone complications and high treatment cost were considered as disadvantages of hormonally manipulated protocols. Natural approaches for endometrium preparation, as a patient-friendly option, have been initiated in recent years. The lack of above disadvantages was the fundamental reason that led to an increasing trend toward this therapeutic approach. However, the natural cycle regimen has a number of controversies regarding its use. Current disadvantages contain a frequent ultrasonic assessment of the follicles, unexpected ovulation and lack of synchronizing development of the endometrium with the dominant follicles (Von Wolff *et al*., 2014; Polyzos *et al*., 2016; Allersma *et al*., 2013; González-Foruria *et al*., 2016; Gordon *et al*., 2013). Thereupon, the evidence is still sparse as to which endometrium preparation protocol is preferred. Some authors recommend considering the patient's preferences, cost-effectiveness, and safety for mother and child (Pennings & Ombelet, 2007; Groenewoud *et al.*, 2012).

In view of the above, we address the pregnancy outcome in modified natural cycles using the HCG regimen versus artificial and mild hormonally stimulated protocols in patients undergoing vitrified thawed blastocyst transfer.

## MATERIALS AND METHODS

This control-randomized trial was carried out at the Fatemezahra Infertility Research Center, affiliated with the Babol University of Medical Science. The study was approved by the Research Ethics committee of Babol University of Medical Science and was registered with the number of 201408021760N36 in the Iran clinical trial registry (IRCT).

### Study population

A total of 131 patients submitted to vitrified thawed blastocyst transfer in our IVF laboratory were invited from March 2015 to January 2016. Women undergoing vitrification thawed blastocyst transfer (VTBT) were eligible for the study when they were normo-ovulatory women, between 20 to 40 years of age, with 19<BMI <30.

The exclusion criteria included women with PCOs, basal FSH>10 IU/ml and basal E_2_ <70 pg/ml, those with untreated thyroid disorders, severe endometriosis, recurrent implantation failure, uterine pathology, recurrent abortion, repeated implantation failure, smokers, athletes and patients who had used any medication in the two previous months that could interfere with the normal function of the hypothalamic-pituitary-gonadal axis.

### Randomization

The 123 women submitted to VTBT, who met the inclusion criteria and provided written informed consent for the study were included. To apply a patient-friendly method, we chose the natural modified cycle with HCG trigger instead of the true natural cycle as the study group. For sample size calculation, we used a confidence interval of 95%, power of 80 in the pregnancy rate between the three groups to choose a sample size of 120 patients - an adequate number in each group to achieve an 80% power of detection at a significant level of 0.05 in a ratio of 1:1:2. The randomization was done at the start of the cycle using sequential numbering based on a computer-generated list that had been prepared at the Statistics Center of the Babol University of Medical Science and sent to us. Then, the participants were randomly assigned to either modified natural cycle with HCG (n=31), mildly hormonally stimulated cycle (n=30) or artificial regimen (n=62). The participant and the infertility expert were not blinded for treatment allocation.

The sonographer was not changed during the procedure. Laboratory and transfer techniques were the same during the procedure.

At first, the patients were assessed by transvaginal ultrasound (TVS) on the third day of men struation (7.5 MHz vaginal probe; Mylab40, Esaote, Italy) to remove the patients with ovarian cysts. Then, a serial TVS measured the endometrial thickness and follicle diameter consistently.

### Outcome measurement

Our primary outcome was the pregnancy rate in the modified natural cycle using the HCG protocol versus the mild hormonally stimulated and artificial protocols of endometrium preparation following vitrified blastocyst transfer. As additional outcome variables, we evaluated the endometrial characteristics in the modified natural cycle versus hormonally stimulated and artificial cycle regimens at the day of vitrified blastocyst transfer.

### Endometrial preparation

We used the natural cycle with HCG for the patients in this group; no medication was administered during the endometrial preparation. The follicles were monitored by TVS until the dominant follicles reached a diameter of 18-20 mm and endometrium thickness >8 mm. Then, 10,000 IU of Human Chorionic Gonadotropin (CG, Daroupakhsh, Iran) was administered for ovulation. Vitrified Blastocysts following warming were transferred after ovulation was observed, usually on 36-38 hours after HCG administration.

The natural cycle with HCG reduces the number of LH monitoring visits required to schedule the day of VTBT; then, we preferred to use HCG for the detachment of the eggs in terms of cost-effectiveness and patient convenience.

The mild hormonally stimulated group with clomiphene citrate (Clomid, Iran Hormone Company) was administered 50 mg daily from day 3 of the menstrual cycle for 5 days. If during TVS a follicle 18-20 mm was visible, ovulation was deemed to have occurred. Then, 10, 000 IU of urinary HCG was administered and the blastocyst were transferred 36-38 hour after HCG.

The Artificial cycles began on the third day of the menstrual cycle or progesterone withdrawal. The dose of oral estradiol valerate (E2) (Aburaihan Pharmaceutical Co., Tehran, Iran) was 2mg bid (4mg/day). A higher initial dose of estradiol (6mg) was administered if the patient showed inadequate endometrial thickness in a previous cycle. TVS was carried out on day 10. If the endometrial thickness reached 8 mm and further, 50mg progesterone was given IM for 3 days (Aburaihan Pharmaceutical Co., Tehran, Iran) and estradiol was continued as well, then the blastocysts were transferred on the fourth day of progesterone administration. If the endometrial thickness was 8mm or less on day 10, the dose of estradiol valerate was increased to 4mg twice/day and the blastocyst were transferred 4-5 days following initiation of progesterone administration if the signs of ovulation were observed upon TVS. If the endometrial thickness did not reach 8 mm up to day 20, or the ovulation was not confirmed, the cycle was cancelled.

For luteal supplementation, vaginal suppository Cyclogest 400mg (Actavis Group, Iceland) twice/daily was recommended for all groups following the day of blastocyst transfer during 14 days.

### Blastocyst transfer

Since the clinical outcomes of vitrification/warming are superior to slow-freezing/thawing (Rienzi *et al*., 2017), we chose the vitrification thawed blastocyst method instead of a frozen embryo or fresh blastocyst.

All participants had blastocyst from their prior cycles, which had been cryopreserved by vitrification and warming by the Cryotop methodology, as per described by Kuwayama (Kuwayama, 2007).

After warming, the blastocyst was partially or completely re-expanded to the dimensions it had before vitrification. We considered a blastocyst had survived after warming if the following morphologic parameters existed; inner-cell mass (ICM) should be equally shaped and sized as before cryopreservation. In addition to the number and cohesiveness of ICM and trophectoderm, and blastocele expansion according to Gardner's criteria (Gardner & Schoolcraft, 1999a). For sample consistency purposes, only good-quality blastocysts were used for transferring. We defined good-morphology blastocysts as the ones that reached at least grades A or B; excellent, (≥3AA) and good, (3, 4, 5, 6, AB and BA) based on ICM and trophectoderm quality score, according to the criteria proposed by Gardner and colleagues (Gardner & Schoolcraft, 1999b).

An embryologist, using the same method, did all laboratory procedures.

It is noteworthy that the eligible women could not be randomized and contributed more than one cycle. Each of the patients received only one good quality blastocyst and the transfer was not repeated if she did not become pregnant. After the transfer, the failed patients were drawn out of the study, and were submitted to another recommended endometrium preparation protocols.

### Outcome measurement

The endometrial maturation was evaluated by the endometrial thickness and the presence of the triple line endometrial pattern at the day of HCG administration in the modified natural and hormonally stimulated cycles and at the day of transferring, and for artificial regimen as well. Endometrial thickness was defined as the maximal distance between the echogenic line of the myometrium and the endometrium that was measured in the midsagittal view by two-dimensional TVS at the day of HCG administration in the modified natural and mild hormonally stimulated cycles, and at the day of transferring for the women submitted to the artificial regimen. Triple-line pattern contains two hypoechoic layers that surrounded a central hyperechoic line.

The duration of endometrial preparation was defined as the interval from the day of menstruation to the day of HCG administration.

The chemical pregnancy test was defined as the serum b-hCG≥30 IU/L, 10 days (two consecutive tests at 2-day intervals) following the blastocyst transfer. The implantation rate was determined by the percentage of gestational sac per blastocyst transferred. A clinical pregnancy was defined as the visualization of a gestational sac with fetal heart activity on TVS in week five of gestation. An ongoing pregnancy was a pregnancy that completed ≥24 weeks of gestation. An abortion was defined as the inability to see a previously confirmed gestational sac or heartbeat between week 7 and week 20.

### Statistical analysis

The statistical analysis was performed with the SPSS (Statistical Package for Social Science, SPSS Inc., Chicago, IL, USA) version 16.00 software. We ran the analysis per protocol and excluded the patients lost to follow up. Therefore, 28 patients in the modified natural cycle, 29 patients in the hormonally stimulated cycle and the 56 patients in the artificial regimen were analyzed. Kolmogorov-Smirnov was used to test the normality distribution of continuous variables. Owing to normally distributed, the statistical comparison was assessed using the ANOVA test for continuous variables, and the Chi-square test was used for categorical variables. Post-hoc test confirms where the differences occurred between groups. The findings were presented by means with standard deviations and the categorical variables were given as percentages (%). *p*-values <0.05 represents statistical significance.

## RESULTS

**Figure 1:**
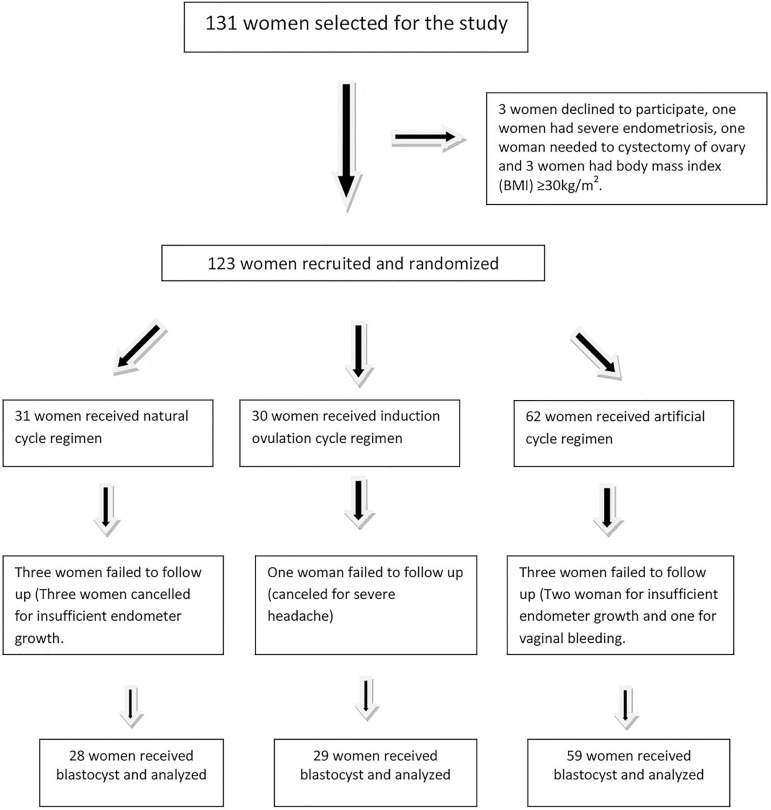
Randomization of the women who participated in the study.

Out of 131 patients eligible for the study, 123 women were recruited according to our exclusion and inclusion criteria, and 8 patients were excluded. The reasons for excluding the participants are illustrated in Figure 1. Finally, the patients were randomized into three groups; natural cycle group (n=31), ovulation induced (n=30), and artificial cycles (n=61).

Three women in the modified natural group and two women in the artificial group were taken off because of insufficient endometrium growth. Finally, three women in the modified natural group, one in the mild hormonally induced group and three women in the artificial group were lost to follow up, hence 28 women in the natural, 29 women in hormonally stimulated and 59 women in the artificial group received blastocysts and entered the study.

As presented in Table 1, the patients in the three groups had similar demographic characteristics. Mean age and BMI in the modified natural group, stimulated ovulation and the artificial group were similar (30.40±4.6 vs. 30.5±5.89 vs. 29.71±3.8, *p*=0.78) (25.82±3.83 vs. 25.36±5.7 vs. 26.19±3.38, *p*=0.77). There were no significant differences among the three groups in regards to infertility duration, infertility cause, type of infertility (primary/secondary) and baseline serum FSH and LH (Table 1).

**Table 1:** Baseline characteristic of the vitrified blastocyst recipients who participated in the study.

Groups	Natural Cycle (28)	Mild hormonally stimulated cycle (29)	Artificial Cycle (59)	*p*-value
**Age (yr) mean±SD**	29.71±3.79	30.31±4.58	30.5±5.59	NS
**BMI(Kg/m^2^) mean±SD**	26.193.24	25.83.29	25.365.27	NS
**Infertility duration (yr) mean±SD**	5.36±3.64	5.83±3.71	6.13±4.4	NS
**FSH ((mIU/mL) mean±SD**	6.19±1.97	7.33±3.44	7.3±2.35	NS
**LH (mIU/mL) mean±SD**	6.17±4.1	5.61±3.63	5.8±3.42	NS
**Cause of infertility n (%)**				NS
**Male**	14 (50)	23 (39)	13 (46.4)	
**Female**	3 (10.7)	17 (28.8)	3 (10.7)	
**Both or other causes**	11 (39.35)	19 (32.2)	12 (42.9)	
**Type of Infertility n (%)**				NS
**Primary**	20 (71.4)	39 (65)	22 (78.6)	
**Secondary**	8 (28.5)	21 (35)	6 (21.4)	

NS: Not significant.

147 vitrified blastocysts were transferred to 116 patients. 36 blastocysts belonged to the natural cycle group, 38 to hormonally stimulated group and 73 to the artificial groups. No significant difference was seen among the women in regards to blastocyst quality in the three groups. All the transferred blastocysts had good morphology, as previously described in the method.

As a whole, 18.64% (22) chemical pregnancies were achieved in 116 blastocyst stage cycles. The gestational sac was not visualized in three patients of the artificial group. The implantation rate was established at 16.1% (19). Fetal heartbeat was declared in 16.1% (19) patients. The total ongoing pregnancy rate was 15.7% (18). One patient in the artificial group had a miscarriage in week 14 of pregnancy.

The findings of pregnancy outcome and endometrial preparation are illustrated in Table 2. No statistically significant differences were found in terms of implantation rate, chemical, clinical, ongoing pregnancy and miscarriage among the three groups.

**Table 2:** Reproductive outcome of the women received vitrified blastocyst.

Groups	Natural (28)	Mild hormonally cycle (29)	Artificial (59)	*p*-value
**Endometrial thickness (mm) (mean±SD) **	8.13±0.95^a^	7.3±1.4^b^	8.34±0.89^c^	<001
**Duration of endometrial preparation (days) mean±SD**	12.68±2.88^d^	11.36±2.43^e^	10.27±1.89^f^	<001
**Blastocyst transferred (n) (mean±SD)**	1.25±0.44	1.32±0.48	1.2±0.4	NS
**Triple line endometrium n (%)**	21 (23.3)	18 (20)	51 (56.7)	NS
**Chemical pregnancy n (%)**	6 (21.43)	4 (13.79)	12 (20.3)	NS
**Implantation rate n (%)**	6 (21.43)	4 (13.79)	9 (15.25)	NS
**Clinical pregnancy n (%)**	6 (21.43)	4 (13.79)	9 (15.25)	NS
**Ongoing pregnancy n (%)**	6 (21.43)	4 (13.79)	8 (13.56)	NS
**Miscarriage n (%)**	0	0	4 (6.8)	NS

a  vs. b: *p*<0.001, b vs. c: *p*<0.02. d vs. e: *p*<0.01, e vs. f: *p*<0.001. NS: Not significant.

*Post hoc* test demonstrated that the endometrium thickness was significantly greater in the artificial vs. the natural cycle using hCG and in natural cycle vs. hormonally stimulated groups, respectively (8.34±0.89 vs. 7.3±1.4, *p*<0.001; 8.13±0.95 vs. 7.3±1.4, *p*<0.02).

## DISCUSSION

In the selected population, we did not find any statistically significant difference in the reproductive outcome of the modified natural cycle with the HCG trigger protocol, the mild hormonally induced cycle or the artificial cycle regimens. However, the results showed a trend towards a slightly higher ongoing pregnancy (7-8% higher), implantation and clinical pregnancy (6-7.5% higher) rates in the modified natural group. To the best of our knowledge, there are a few randomized trials that investigated the three mentioned cycle regimens for endometrium preparation simultaneously; however, our results are consistent with those of previous studies involving clinical outcomes of naturally endometrial preparation compared either with artificial, or hormonally stimulated cycles (Hancke *et al*., 2012; Kim *et al*., 2010; Kyrou *et al*., 2010).

Although the natural protocol showed non-significant higher implantation rate versus hormonally stimulated cycle and artificial protocol (21% *vs.* 13% and 15%), it was associated with less miscarriage (0 and 0 *vs.* 6.8%). It seems that the natural cycle is at least safe and lacks consequences in comparison to the other cycle regimens mentioned; besides, one has to consider patient convenience, preference and cost-effectiveness. This outcome is contrary to that of Chang *et al.* who found greater miscarriage rates in the natural cycle regimen using HCG versus artificial cycle. This inconsistency may be because Chang selected the samples' cycle regimen according to patient convenience and cost in their retrospective study, and we recruited the patients randomly (Chang *et al*., 2011).

Implantation is a multifactorial phenomenon, requiring synchronization between the developing embryo and optimal endometrial environment (Lee *et al*., 2006). To improve implantation rates, some authors have propounded optimal embryo conditions with the natural protocol. To achieve a better outcome, Chang suggested transferring the vitrified blastocyst to a natural endometrium preparation (Chang *et al*., 2011). Xiao proposes that the natural cycle is superior to reproductive outcome in comparison to artificial cycle when excellent embryo conditions are met. For sample consistency, we decided to transfer excellent or good quality blastocysts for all the cycle regimens (Xiao *et al*., 2012).

Our other important statistically relevant finding was that the mean endometrial thickness on the day of progesterone initiation or hCG administration was more frequently found in the artificial and natural cycle groups than the mild induced cycle group; however, the frequency of triple endometrial patterns in the three groups were comparable. We expected higher endometrial thickness in the artificial cycles owing to greater Estradiol levels in such cycles. Chang concluded that this alternation also occurs in the natural cycle due to the decidualization influence of HCG on the endometrium during the implantation (Chang *et al*., 2011). In our study, this might be a possible explanation for a high endometrium thickness in the natural cycle group. Nevertheless, we found no association between reproductive the outcome and endometrium thickness or triple line pattern in the three groups. As we did not eliminate some confounding variables, maybe these results need to be interpreted with caution.

Our findings are in accord with those from Yoeli *et al.* (2004) indicating that no relationship was found between decreased implantation or pregnancy rates and increased endometrial thickness in assisted reproduction. The present study raises the possibility that other factors, including patient's convenience and request, social status, and physician's preferences may be considered in the choice between these three endometrium preparation protocols.

Maybe, a weakness of our study is the lack of blindness that may cause potential biases. In addition, we compared endometrial features on the day of progesterone or HCG administration amongst three cycle regimens. Whereas, the endometrium growth continues to the day of blastocyst transfer, may be the endometrial characteristics in the transfer day is not exhaustive. In addition, the number of patients in each of the groups was small; and with small sample size, caution must be applied, as the result might not be a source of entire certainty. Our findings must be elucidated by well-conducted RCTs with large-scale and controlled variables design.

## CONCLUSION

The patients with normal ovarian function achieved the desired outcome using natural with HCG as well as mild hormonally and artificial cycles. We recommend natural protocol with HCG trigger as a therapeutic alternation for preparation of endometrium prior to vitrified thawed blastocyst transfer.
